# Team Handball Experts Outperform Recreational Athletes in Hand and Foot Response Inhibition: A Behavioral Study

**DOI:** 10.3389/fpsyg.2019.00971

**Published:** 2019-05-07

**Authors:** Holger Heppe, Karen Zentgraf

**Affiliations:** ^1^Institute of Sport and Exercise Sciences, Human Performance and Training, University of Münster, Münster, Germany; ^2^Otto Creutzfeldt Center for Cognitive and Behavioral Neuroscience, University of Münster, Münster, Germany; ^3^Department of Movement Science and Training in Sports, Institute of Sport Sciences, Goethe University Frankfurt, Frankfurt, Germany

**Keywords:** response inhibition, effector specificity, motor expertise, handball, two-choice RT, motor inhibition

## Abstract

Inhibition is a central component of human behavior. It enables flexible and adaptive behavior by suppressing prepotent motor responses. In former studies, it has been shown that sport athletes acting in dynamic environments exhibit superior motor inhibitory control based on sensory stimuli. So far, existing studies have corroborated this in manual motor response settings only. Therefore, this study addresses the effector specificity of the inhibition benefit in elite athletes compared to physically active controls. A sport-unspecific stop-signal task has been adapted for hand as well as feet usage and 30 elite handball players as well as 30 controls were tested. A repeated-measures ANOVA with the two factors “effector” (hands, feet) and “group” (expert, recreational athletes) was conducted. Our results suggest no group differences in two-choice response times, but a convincing superiority of handball players in inhibitory control (i.e., shorter stop-signal reaction times), predominantly when responding with their hands, with weaker differential effects when responding with their feet. This suggests that motor inhibition might be a comprehensive performance characteristic of sport athletes acting in dynamic environments, detectable predominantly in eye-hand coordination tasks.

## Introduction

Imagine you stand on a soccer field in an offensive play and receive a pass in the midfield from your teammate. Based on your visual scanning of the court, you decide to play a through pass to your starting teammate, but when initiating the movement to play the pass, you suddenly recognize that the defender anticipated your decision and closed the passing lane right in time. If you are able to cancel the execution of your passing action, you avoid a misdirected pass and loss of ball possession. Obviously, athletes in interactive sports have to respond quickly to their teammates’ or opponents’ actions and, equally important, frequently inhibit their already initiated responses (e.g., when reacting to feints or when the previously opened passing lane is suddenly closed). There is some recent evidence that athletes acting in dynamic sports environments are superior in inhibiting their motor responses based on sudden perceptual input, both for younger (Verburgh et al., 2014; Huijgen et al., 2015) and adult athletes (Di Russo et al., 2006; Wang et al., 2013; Zhang et al., 2015; Brevers et al., 2018). Specifically, superior inhibitory control is indicated by shorter stop-signal reaction times (SSRTs), meaning that athletes need less time to withhold their prepotent response. The stop-signal task requires study participants to process two types of signals, a visual go-signal and a quick motor response; and a visual stop-signal, presented with varying delays after the go-signal, asking participants to withhold their prepared motor response. In most studies, athletes’ benefits have been shown when the sensory inhibition signal is visual or acoustic, when the motor response (or not-response) is required manually (i.e., pressing buttons) and when athletes used manual actions and tools for their sports expertise (e.g., Taekwondo and fencing athletes in Brevers et al., 2018; tennis players in Wang et al., 2013; fencers in Zhang et al., 2015). In all these sports, however, motor actions with lower extremities such as sprinting and stopping after a run, change-of-direction skills, jumping and delaying the jump, preparing and retarding an explosive step forward, reacting or not-reacting to feints etc., are also essential for elite performance. Indeed, motor performance superiority related to lower extremities for elite athletes in jumping, sprinting, tapping etc., compared to non-athletes is well established and among others, linked to greater muscular strength in sports athletes (Suchomel et al., 2016, for review).

The existing literature also suggests that this lower extremities’ power and strength underlies superior performance in general sport skills (e.g., jumping, sprinting, change-of-direction; Nimphius et al., 2010) as well as sport-specific skills. For example, jump shooting is the most frequent throw in basketball. The higher the release point of the ball, the harder the jump is to block by a defender. The jump height to reach this height is indirectly represented by the power of the lower limbs (Struzik et al., 2014).

An open question therefore is the motor-effector specificity concerning the superiority in inhibitory control in elite athletes. Thus, this present study investigated whether response inhibition is effector-specific (i.e., hands, feet) and how this is related to motor expertise. Based on the fact that lower leg and feet motor performance is essential for elite sport-games athletes, we hypothesized a non-effector-specific superiority in inhibitory control for elite handball players compared to recreational athletes. To address this aim, participants performed the stop-signal task not only manually, but also with their feet. We hypothesized shorter SSRTs in athletes for hands and for feet compared to physically active, but not specifically trained controls.

## Methods

### Participants

Male professional handball players playing in the second league in Germany, from three different clubs (*n* = 30, mean age 24.2 years ± 4.6) and male recreational non-handball athletes (*n* = 30, mean age = 23.2 years ± 4.6) were tested in a response-inhibition paradigm created by Verbruggen and Logan (2008). Inclusion criteria for the control group were: male, no high expertise level in any team sports, physically active. The recreational athletes stated following sports as their main sport: soccer (*n* = 13), gym training (*n* = 6), volleyball (*n* = 4), track and field (*n* = 3), basketball (*n* = 2), tennis, karate and table tennis. The participants who played in a league system played in the lower third of leagues in Germany.

We assessed the level of physical activity of control group participants with the short form of the International Physical Activity Questionnaire (Craig et al., 2003), which assesses vigorous-intensity activity, moderate-intensity activity and walking to compute an overall score. The levels of physical activity of the control group can be categorized into moderately and highly physically active (*M* = 4890 MET-minutes per week ± 1956, *Mdn* = 4491, range from 2292 to 9678). These totals were achieved via 2512 ± 1540 MET-minutes per week of vigorous activity (*Mdn* = 2880, range from 0 to 6720), 1458 ± 1199 MET-minutes per week of moderate-intensity activity (*Mdn* = 1080, range from 0 to 5040) and 920 ± 1163 MET-minutes per week of walking activity (*Mdn* = 528, range from 0 to 4158).

### Power Analysis

Three studies were identified to inform the power analysis. All of them showed superior performance of experts in interactive sports: Effects were *d* = 0.89 (Verburgh et al., 2014), *d* = 0.53 (Huijgen et al., 2015) and *d* = 1.2 (Wang et al., 2013).

We decided to use the effect size of Verburgh et al. (2014) as a basis of a power analysis for the main dependent variable stop-signal reaction time (SSRT) because it was based on the largest sample size. Uncertainty of the effect-size estimate was considered using the lower boundary of the 60% confidence interval (via the MBESS R package; Kelley, 2016) with 84 experts and 42 controls (Perugini et al., 2014). As a result, we obtained an estimate of the effect size of *f* = 0.33. Alpha-level was set to 0.05 and power level to *P* = 0.80. Therefore, 58 participants are needed in this within-between 2×2 design (Faul et al., 2007).

### Dependent Measures

Response inhibition was estimated via a SSRT test developed by Verbruggen et al. (2013) with a go- and a stop-condition. In the go-condition, participants had to react to a go stimulus (left or right arrow) with the left or right hand/foot as fast as possible. In the stop-condition (25% of the trials), the arrow turned blue after a variable delay (stop-signal delay, SSD). In this case, participants had to inhibit their planned response. The stimuli (288 trials) were presented until an answer (right or left button push) was given. The experiment was programmed adaptively. The delay between stimulus onset and stop signal was variable in steps of 50 ms. We used an adaptive staircase procedure, that means if the participant inhibits successfully, the following SSD in a stop-signal trial gets 50 ms longer and the task gets harder. If the participant fails to inhibit, the following SSD gets 50 ms shorter, and the task gets easier. This results in testing around the individual threshold and an inhibition success rate of usually about 50%. The difficulty in measuring response inhibition is that the time when the participant is not reacting (i.e., successful inhibiting) has to be recorded. Therefore, an estimation method has to be used. We used the integration method. Die dependent variable SSRT is estimated by subtracting the mean SSD from the mean response time in the go-condition. The mean response time is calculated by rank-ordering all RTs and selecting the *n*th go RT, where *n* equals the number of trials (*n* = 288) multiplied by the error rate. For example, if a participant has an error rate of 60%, the 173rd (288*0.6) response is selected as the go-RT. To estimate the SSRT for a participant, the arithmetic mean of the SSDs is subtracted from this go-RT.

In response-inhibition tasks, it is important not to slow down and to “wait” for the stop signal. To encourage participants not to slow down their go-response times and follow the instruction to react as fast as possible, we made three changes to the default settings proposed by Verbruggen et al. (2008). The first change was to give a German instruction and use visualization to describe the following experiment. The second change was to set the initial SSD from 250 to 100 ms. This led to a higher share of successfully canceled actions at the beginning since trials with shorter SSDs are easier. The third change related to tests of two-choice response times (the go-condition in a stop-signal task) prior to the stop-signal task. Before the response inhibition experiment started, two-choice response times (2-CRTs) were measured. The participants were told to react as fast and accurately as possible to the direction of the arrow with feet or hands, in counterbalanced order. After eight test trials for hands and feet, respectively, 48 trials of feet and hands were measured. After the acquisition of 2-CRTs, participants received the instruction that they still have to respond as fast and accurately as possible, but occasionally, the arrow is going to turn blue. If this was the case, they were instructed to try to withhold their response. They were told both orally and in writing that the task difficulty is adaptive and that it is going to be very hard, well-nigh impossible to withhold the response in about half of the trials.

Besides the advantage of encouraging participants to react with maximum speed, the preceding 2CRT test allowed to test whether there is a difference in isolated 2-CRTs between both groups and to quantify how much participants slow down their go-responses due to strategic considerations (or due to the uncertainty whether a following stimulus is a go- or stop-trial). The experiment file can be found on https://osf.io/cbtz6.

### Data Analysis

The pre-planned analysis followed the analysis script of Verbruggen et al. (2008). We used the integration method to estimate the dependent measure SSRT (Verbruggen et al., 2013). A repeated-measures ANOVA with the two factors “effector” (hands, feet) and “group” (experts, recreational athletes) was conducted. Since we hypothesized that handball experts perform better than recreational athletes, *post hoc* tests were performed one-tailed. We hypothesized an expert advantage both in the hand and the foot condition. Since all previous studies we found on this topic tested response inhibition with finger movements, we had a strong hypothesis for differences in the hand condition with superior performance of expert athletes. Regarding the foot condition, we knew that there is a medium-sized correlation between hands and feet SSRTs from an own pilot study, which should also lead to an effect in the same direction. The second reason we expected an expert advantage in the foot condition was the importance of lower-body performance in attack and defense, even in a (when on-the-ball actions are put in focus) hand-dominant sport like handball. However, our previous knowledge about feet response inhibition was not broad enough to expect a group difference in the foot condition somewhere as large as in the hand condition. Based on previous reaction time studies including reaction tests with feet and hands (Montés-Micó et al., 2000; Pfister et al., 2014), we expected better performance in the hands condition.

When performing additional Bayesian analyses, we reported the Bayes Factor and followed the recommendations by Wetzels and Wagenmakers (2012) to interpret the grade of evidence. We used the statistical software (JASP Team, 2018, version 0.8.3.1) for descriptive, Frequentist and Bayesian statistics and the “ggplot2” R package (Wickham, 2016) for visualization. Supplementary materials and raw data can be found at https://osf.io/cbtz6.

## Results

### Two-Choice Response Times

**FIGURE 1:**
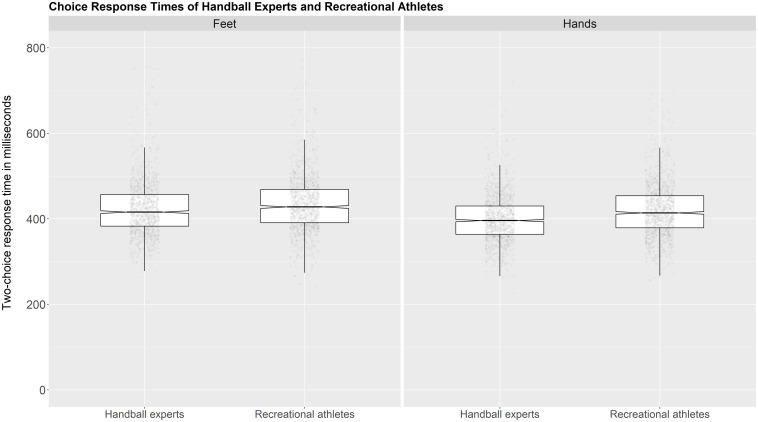
Two-choice response times of handball experts (*n* = 30) and recreational athletes (*n* = 30) with feet and hands. Each data point represents one response time (only correct trials are plotted). The box shows the interquartile range (25th to 75th percentile within the box). The length of the whiskers is 1.5 × interquartile range. The line in the middle shows the median RT, which is surrounded by the notch. The notch represents a confidence interval that is based on 1.57 × (interquartile range/sqrt of n). Figure available at http://bit.ly/2crt-hb under CC license https://creativecommons.org/licenses/by/4.0/.

Results show a significant main effect of response effector [*F*_(1,58)_ = 24.98, *p* < 0.001, $\eta {}_{p}^{2}$ = 0.301] with shorter two-choice response times (2-CRTs) in the hands (*M* = 408.02 ms, *SD* = 36.03) compared to the feet condition (*M* = 423.58 ms, *SD* = 28.87). The main effect of group [*F*_(1,58)_ = 3.06, *p* = 0.085, $\eta {}_{p}^{2}$ = 0.05) was not significant as well as the interaction between response effector and group [*F*_(1,58)_ = 2.30, *p* = 0.134, $\eta {}_{p}^{2}$ = 0.038] (see Figure 1).

A Bayesian analysis was conducted to give further information on response effector and group differences. Individual comparisons based on a *t*-test with a Cauchy prior (center = 0, *r* = 0.707) showed a BF01 of 3.75 for the response-effector (moderate evidence for no difference between hands and feet) and a BF01 of 0.55 (anecdotal evidence for no group difference) for the group comparison.

### Response Inhibition

Results showed a significant main effect of response effector [*F*_(1,58)_ = 26.54, *p* < 0.001, $\eta {}_{p}^{2}$ = 0.314] with shorter SSRTs in the hands compared to feet. Also, a significant main effect of group was evident with shorter SSRTs in the handball experts [*F*_(1,58)_ = 7.89, *p* = 0.007, $\eta {}_{p}^{2}$ = 0.120]. The interaction between response effector and group was not significant [*F*_(1,58)_ = 2.87, *p* = 0.096, $\eta {}_{p}^{2}$ = 0.047].

**FIGURE 2:**
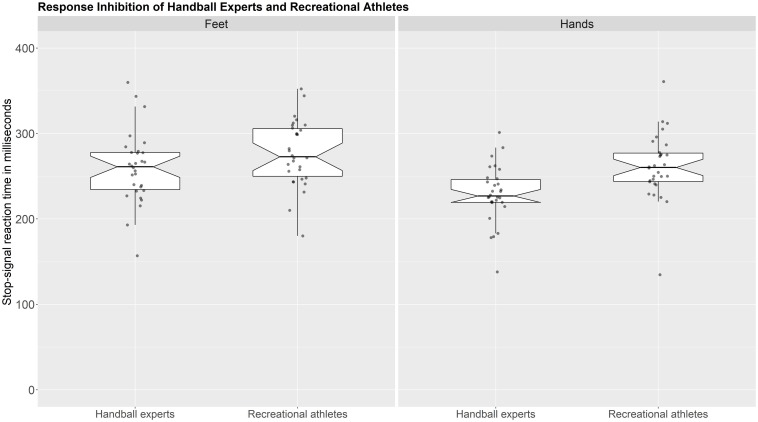
Response inhibition of handball experts (*n* = 30) and recreational athletes (*n* = 30) with feet and hands. Shorter SSRTs indicate higher inhibitory control. Each data point represents the SSRT of one participant. The box shows the interquartile range (25th to 75th percentile within the box). The length of the whiskers is 1.5 × interquartile range. The line in the middle shows the median SSRT, which is surrounded by the notch. The notch represents a confidence interval that is based on 1.57 × (interquartile range/sqrt of n). Figure available at http://bit.ly/ssrt-hb under CC license https://creativecommons.org/licenses/by/4.0/.

To further elaborate on the reported main effects, planned one-tailed *t*-tests in the feet condition showed no significant difference between handball experts (*M* = 259.5 ms, *SD* = 41.6) and recreational athletes (*M* = 276.74 ms, *SD* = 38.4), *t*(58) = 1.67, *p* = 0.10, 95% CI (−0.08, 0.943), *d* = 0.43. However, in the hands condition, a planned one-tailed *t*-test showed a significant difference between handball experts (*M* = 229.36 ms, *SD* = 32.83) and recreational athletes [*M* = 261.52 ms, *SD* = 39.00), *t*(58) = 3.46, *p* = 0.001, 95% CI (0.362, 1.423), *d* = 0.89]. Individual group comparisons based on a Bayesian independent samples *t*-test under a one-sided hypothesis (superiority of handball experts) with an informed Cauchy prior (center = 0.5, *r* = 0.5) showed a BF10 of 85.72 for the hands condition (suggesting very strong evidence for a group difference) and a BF10 of 1.64 for the feet condition (suggesting anecdotal evidence for a group difference). A Bayes Factor robustness check shows no meaningful differences in the interpretation of evidence between different prior widths within a plausible range for both conditions. As an example, we computed the same analysis with a default Cauchy prior (center = 0, *r* = 0.707). It showed a BF10 of 60.01 for the hands condition (suggesting very strong evidence for a group difference) and a BF10 of 1.46 for the feet condition (suggesting anecdotal evidence for a group difference). Results of both groups in both conditions are presented in Figure 2.

Stop-signal reaction times of hands and feet were positively correlated, Pearson’s *r*(58) = 0.63, 95% CI (0.48, 1.00), *p* < 0.001 (see Figure 2). There was no significant difference (*p* = 0.224) in the hands-feet correlation between handball experts [Pearson’s *r*(29) = 0.67, 95% CI (0.41, 0.83)] and recreational athletes [Pearson’s *r*(29) = 0.54, 95% CI (0.22, 0.75)].

### Additional Analysis

To check to what extent participants slowed down their responses in the stop-signal paradigm and whether there are group differences, we did some further analysis. First, we calculated the “slowing down” of participants’ response times: we calculated the mean of the no-signal response times (ns-RT: the response time, when no stop signal occurred) in the stop-signal reaction time task and the mean of the response time in the two-choice response time task (2-CRT). To calculate a measure for slowing (slowing index), ns-RT (the average response time in the response-inhibition experiment on no-signal trials) was divided by 2-CRT from the first part of the experiment. Results show that participants on average slowed down their response in both conditions by 29.8% when they use their hands and 23.9% when they use their feet (Table 1).

**TABLE 1:** Slowing index of both effectors.

	**Hands**	**Feet**
Mean slowing index*	1.298	1.239
Std. deviation	0.259	0.229
Minimum	0.955	0.712
Maximum	2.058	2.047

*Slowing index, Average response time in the two-choice response time task/average response time in the response inhibition experiment on no-signal trials.

Slowing in response times was positively correlated to shorter SSRTs, indicating an effect of strategy on the dependent measure. More “slowing down” led to higher mean response times in the no-signal trial conditions, but this negative effect on SSRTs was overcompensated by longer SSDs. To see whether there is a group difference in slowing (e.g., strategies), the slowing index was compared via Bayesian independent *t*-tests with a Cauchy prior (center = 0, *r* = 0.707) for both effectors. In the feet condition, a BF01 of 2.335 gives anecdotal evidence for no group difference. In the hands condition, a BF01 of 1.163 gives anecdotal evidence for no group difference, either.

## Discussion

Previous studies have suggested specific perception-action performance benefits of elite athletes; relevantly, athletes need less time for canceling a prepotent motor response. The aim of the current study was to elucidate whether this expertise effect extends to different bodily effectors. Accordingly, we tested athletes and physically active controls when performing tests in inhibitory control with their hands as well as their feet. The main finding of this study is that when responding with their hands, handball experts showed substantial shorter SSRTs, indicating superior response inhibition, compared to recreational athletes (*d* = 0.89, BF10 = 60.03). When responding with their feet, the difference between groups decreased (*d* = 0.43, BF10 = 1.46). The Bayes factors provide strong evidence of an advantage of handball athletes when using their hands (suggesting that these data are 60 times more likely to be observed under the alternative hypotheses) and anecdotal evidence for an expert advantage when using their feet in a response inhibition task. Noteworthy, the two groups did not differ in two-choice response time measures, which has sometimes been suggested to be a sports-expertise characteristic when using sport-specific or sports-related stimuli (Mori et al., 2002; Voss et al., 2010; Heppe et al., 2016). Based on the quasi-experimental, cross-section design of this study, it might be possible that individuals with enhanced motor inhibitory control select sports as their field, however, our data suggest that athletes’ superiority is not related to reacting faster to a stimulus *per se*. It might speak for an isolated expertise effect and the relevance of cancelation of initiated responses in interactive sports such as handball. The frequent use of hands is an important feature in interactions in handball (ball catching, throwing, defending, etc.), however, our data do also not explain whether this effect is due to training. Additionally, at the moment it is not known how and whether this inhibitory skill develops during the lifespan. We will address these open questions in future studies.

Although there is a differential effect for the effectors, the medium-sized positive correlation of SSRTs of hands and feet in both groups might speak for a common control mechanism for both effectors. The suggestion of a central-control response-inhibition mechanism is supported by a study that shows a negative correlation between degree of damage of the inferior frontal gyrus and performance in a response-inhibition test (Aron et al., 2003). More evidence is provided by a study showing an impairment of response inhibition when transcranial magnetic stimulation of the inferior frontal gyrus was applied (Chambers et al., 2006) and by a study suggesting that participants with ADHD perform worse in response-inhibition tests compared to age-matched controls (Senderecka et al., 2011).

A limitation of this study is the lack of a foot-dominant group. A possible approach for a constructive replication could be a design with amateur and professional soccer players and amateur and professional handball or basketball players. This could give clearer insights on the influence of expertise and of hand respective foot dominance on response inhibition performance of both effectors.

## Conclusion

In conclusion, this study supports existing literature for enhanced inhibitory control in athletes of interactive sports. Evidence for a general effect for different effectors, however, is sparse. This is noticeable since some studies suggest a central mechanism for inhibitory control and expertise in interactive sports encompasses motor skills from lower extremities, too. Whether interactive sports training with the need to repeatedly process information in highly dynamic environments improves inhibitory control should be clarified in further studies, just as why eye-hand coordination could benefit more than eye-foot coordination.

## Ethics Statement

All subjects gave written informed consent in accordance with the Declaration of Helsinki. The protocol was approved by the local ethics committee of the University of Münster.

## Author Contributions

HH and KaZ designed the experiments, analyzed and interpreted the data, edited the manuscript, and approved the final version to be published and are accountable for all aspects of the work. HH performed the experiments and wrote the initial draft of the manuscript.

## Conflict of Interest Statement

The authors declare that the research was conducted in the absence of any commercial or financial relationships that could be construed as a potential conflict of interest.
